# Whole-genome resequencing of *Osmanthus fragrans* provides insights into flower color evolution

**DOI:** 10.1038/s41438-021-00531-0

**Published:** 2021-05-01

**Authors:** Hongguo Chen, Xiangling Zeng, Jie Yang, Xuan Cai, Yumin Shi, Riru Zheng, Zhenqi Wang, Junyi Liu, Xinxin Yi, Siwei Xiao, Qiang Fu, Jingjing Zou, Caiyun Wang

**Affiliations:** 1 Hubei Engineering Research Center for Fragrant Plants, Hubei University of Science and Technology, Xianning, 437100 China; 2 Xianning Research Academy of Industrial Technology of Osmanthus fragrans, Xianning, 437100 China; 3 Key Laboratory of Horticultural Plant Biology, Ministry of Education, Huazhong Agricultural University, Wuhan, 430070 China; 4 Xianning Vocational Technical College, Xianning, 437100 China; 5 Xianning Forestry Academy of Sciences, Xianning, 437100 China; 6 Wuhan Frasergen Bioinformatics Co., Ltd., Wuhan, 430070 China

**Keywords:** Plant sciences, Genetics

## Abstract

*Osmanthus fragrans* is a well-known ornamental plant that has been domesticated in China for 2500 years. More than 160 cultivars have been found during this long period of domestication, and they have subsequently been divided into four cultivar groups, including the Yingui, Jingui, Dangui, and Sijigui groups. These groups provide a set of materials to study genetic evolution and variability. Here, we constructed a reference genome of *O. fragrans* ‘Liuyejingui’ in the Jingui group and investigated its floral color traits and domestication history by resequencing a total of 122 samples, including 119 *O. fragrans* accessions and three other *Osmanthus* species, at an average sequencing depth of 15×. The population structure analysis showed that these 119 accessions formed an apparent regional cluster. The results of linkage disequilibrium (LD) decay analysis suggested that varieties with orange/red flower color in the Dangui group had undergone more artificial directional selection; these varieties had the highest LD values among the four groups, followed by the Sijigui, Jingui, and Yingui groups. Through a genome-wide association study, we further identified significant quantitative trait loci and genomic regions containing several genes, such as ethylene-responsive transcription factor 2 and Arabidopsis pseudoresponse regulator 2, that are positively associated with petal color. Moreover, we found a frameshift mutation with a 34-bp deletion in the first coding region of the carotenoid cleavage dioxygenase 4 gene. This frameshift mutation existed in at least one site on both alleles in all varieties of the Dangui group. The results from this study shed light on the genetic basis of domestication in woody plants, such as *O. fragrans*.

## Introduction

Sweet osmanthus (*Osmanthus fragrans* Lour.), belonging to the family Oleaceae, is a well-known ornamental germplasm native to the Sino-Himalayan region^1^. It has been cultivated in China for more than 2500 years. More than 160 cultivars of *O. fragrans* have been classified based on phenotypes, such as flower color and blooming season. They have been divided into four cultivar groups, including the Yingui group (Albus group), which has white to pale yellow flowers; the Jingui group (Luteus group), which has yellow flowers; the Dangui group (Aurantiacus group), which has orange/red flowers that bloom mainly in autumn for commercial harvest; and the Sijigui group (Asiaticus group), which has pale yellow to yellow flowers that bloom throughout most of the year^2–4^. It is thought that varieties in the Sijigui group and Yingui group are less differentiated from wild *O. fragrans* than the other two groups, which probably originated earlier^2^. The results of microsatellite marker analysis indicate that the varieties in the Jingui and Dangui groups, which displayed more significant genetic differentiation, might have diverged earlier^4^. Thus, the evolutionary relationships of varieties with different colors are still not clear.

Due to their ornamental and commercial value, flowers have long been a focus of interest in the study of *O. fragrans*. It has been reported that α-ionone and β-ionone are the main floral components of *O. fragrans*^5–7^. The accumulation of α-ionone and β-ionone in the cultivars of the Yingui, Jingui, and Sijigui groups is higher than that in the Dangui group, mainly due to the higher efficiency of carotenoid cleavage^8,9^. Furthermore, the presence of white, yellow, and orange color varieties is primarily attributable to the level of carotenoids, whereas flavonoids are speculated to provide only the background color^10^. Thus, the main differences in flower color and floral fragrance among varieties in different groups of *O. fragrans* are determined mainly by the degree of carotenoid accumulation and cleavage. Carotenoid cleavage dioxygenase 1 (*CCD1*) and *CCD4* are crucial contributors to the cleavage of α-carotene and β-carotene into α-ionone and β-ionone^9,11^. The most critical factor determining the diversity of carotenoid concentrations was the differential expression level of *CCD4*^10,12^. This leads to the question, what role does the *CCD4* gene play in the evolution of *O. fragrans* flower color?

More recently, genome sequencing of *O*. *fragrans* ‘Rixianggui’ (OFR) in the Sijigui group, which blooms for most of the year, has been performed at the chromosome level^13^. However, a systematic study to chart the genetic architecture of ornamental traits in a large population using a genome-wide association (GWA) method has not yet been performed. As most cultivars of *O*. *fragrans* bloom in autumn, we generated a reference genome for *O. fragrans* ‘Liuyejingui’ (OFL) from the Jingui group. In addition to the flowering time, OFR has fewer flowers at each blooming event, with a typical complete pedicel and pale yellow flower color. OFL produces many flowers that typically bloom for a week twice per year on average; the flowers are lemon yellow in color, with a strong fragrance and high essential oil contents, and are harvested for ornamental use as well as food and industrial uses^14,15^. We also reported on genomic variations and population evolution by resequencing 119 *O*. *fragrans* accessions with different colors from the four groups. We further sequenced the transcriptomes of different tissues of OFL, such as the rhizomes, leaves, flowers, and flowers, in different flowering stages to validate the quantitative trait loci (QTLs) and functional *CCD4s* through the expression of candidate genes between transcriptomes. For the first time, the present study explains the origin and evolutionary relationship of varieties in different groups of *O. fragrans* and color formation in the different varieties in terms of the deletion of the *CCD4* gene structure.

## Materials and methods

### Plant materials

For genome sequencing, leaf samples were collected from OFL on the campus of Huazhong Agricultural University (Wuhan, China) (114°21′ W, 30°29′ N). For resequencing, leaves were collected from 119 representative *O. fragrans* landraces and three close relatives of *osmanthus*, including *O. cooperi*, *O. × fortunei*, and *O. heterophyllus* (G. Don) P. S. Green var. *Heterophyllus* (Supplementary Table 1).

### Genome sequencing and resequencing

Fresh, healthy leaves were harvested from the best-growing individuals and immediately frozen in liquid nitrogen, followed by preservation at −80 °C in the laboratory prior to DNA extraction. High-quality genomic DNA was extracted using a modified Cetyltrimethyl Ammonium Bromide method^16^. For genome sequencing, single-molecule real-time (SMRT) libraries were constructed and sequenced using a PacBio Sequel II instrument (Pacific Biosciences, Menlo Park, CA, USA) at Frasergen Bioinformatics Co., Ltd. (Wuhan, China). For resequencing, 1 μg DNA per sample was used as the input material, and sequencing libraries were generated using the VAHTS Universal DNA Library Prep Kit for MGI (Vazyme, Nanjing, China) following the manufacturer’s recommendations. Library quantification and size measurement were performed using a Qubit 3.0 Fluorometer (Life Technologies, Carlsbad, CA, USA) and a Bioanalyzer 2100 system (Agilent Technologies, CA, USA). Subsequently, libraries of 122 accessions were constructed and sequenced on an MGI-SEQ 2000 platform at Frasergen Bioinformatics Co., Ltd.

### Transcriptome sequencing

To obtain information that assists in the annotation of genes, the Iso-Seq method was performed to produce full-length transcripts using SMRT sequencing^17^. RNA was prepared from flowers, leaves, stems, and roots collected from the same tree and processed for library construction. Total RNA was extracted using TRIzol reagent (Invitrogen) according to the manufacturer’s protocol. RNA-seq libraries were prepared using the Clontech SMARTer cDNA synthesis kit according to the manufacturer’s recommendations and were then sequenced on the MGI-SEQ 2000 platform at Frasergen Bioinformatics Co., Ltd. and Igenebook Bioinformatics Institute (Wuhan, China).

### Genome assembly

PacBio SMRT sequencing technology and a high-throughput chromatin conformation capture (Hi-C)-based scaffolding method were used to perform chromosome-level assembly of the OFL genome. With one SMRT cell in the PacBio Sequel platform, we generated 174.53 Gb subreads by removing adaptor sequences within sequences. The longest 150X subread data were used for the genome assembly of *O. fragrans*. The initial assembly results were generated by using the default parameters of the mecat2 tool with the longest 150X subread data. To correct errors in the primary assembly, we used the Racon (v1.3.1)^18^ pipeline to refine the genome. Finally, we used Illumina-derived short reads to correct any remaining errors by Pilon (v1.22)^19^. The short reads from the Illumina platform were quality filtered by HTQC (v1.92.310)^20^.

For anchored contigs, clean read pairs generated from the Hi-C library were mapped to the polished OFL genome using BWA (bwa-0.7.17). Paired reads with mates mapped to a different contig were used to perform Hi-C-associated scaffolding. Contigs were then successfully clustered into 23 groups with the agglomerative hierarchical clustering method in Lachesis^21^. Lachesis was further applied to order and orient the clustered contigs.

### Annotation of repetitive sequences

Two methods were combined to identify the repeat contents in our genome: homology-based analysis and de novo prediction. With homology-based analysis, we identified the known transposable elements (TEs) within the OFL genome using RepeatMasker (open-4.0.9)^22^ with the Repbase TE library^23^. RepeatProteinMask searches were also conducted using the TE protein database as a query library. By de novo prediction, we constructed a de novo repeat library of the OFL genome using RepeatModeler, which automatically executed two core de novo repeat-finding programs, RECON (v1.08)^24^ and RepeatScout (v1.0.5)^25^. Furthermore, we performed a de novo search for long terminal repeat (LTR) retrotransposons against the OFL genome sequences using LTR_FINDER (v1.0.7)^26^. We also identified tandem repeats using the Tandem Repeat Finder package^27^ and noninterspersed repeat sequences, including low-complexity repeats, satellites and simple repeats, using RepeatMasker. Finally, we merged the library files of the two methods and used RepeatMaker to identify the repeat contents.

### Annotation of protein-coding gene

We predicted the OFL genome’s protein-coding genes using three methods: ab initio, homology-based and RNA-seq predictions. We used Augustus (v3.3.1)^28^ and Glimmer^29^ to perform ab initio gene prediction. Exonerate (v2.2.0, -model protein2genome-showtargetgff 1)^30^ GeneWise (2.4.1, -trev -genesf -gff -sum)^31^, and Solar (0.9.6, a prot2genome2 -n 200000 -z -f m8)^32^ were used to conduct homology-based gene prediction. To carry out RNA-seq-aided gene prediction, we first assembled clean RNA-seq reads into transcripts using TopHat (v2.1.1)^33^, and the gene structure was formed using Cufflinks (v2.2.1, -I 300000 -p 4 -L CUFF4)^34^. To obtain a more complete gene structure, we also used Iso-seq data. First, the sequencing data were made redundant by CD-HIT (v4.6.7, -AL 1000 -AS 100 -G 0 -M 2500 -aL 0.85 -aS 0.98 -c 0.98 -T 15)^35^. Then, the reference genome was compared with GMAP (v2018-07-04, -n 5 -min-intronlength = 9 --max-intronlength-middle = 200000 -t 15 -A -f 2)^36^. Finally, TransDecoder (v5.3.0, default) (http://transdecoder.sourceforge.net/) structure prediction was performed. Finally, Maker (v3.00)^37^ was used to integrate the three methods’ prediction results to predict the genes.

Gene functions were inferred according to the best match of the alignments to the National Center for Biotechnology Information (NCBI) non-redundant, TrEMBL^38^, InterPro^39^. Swiss-Prot^38^, and Kyoto Encyclopedia of Genes and Genomes (KEGG) databases^40^ using BLASTP (NCBI BLAST v2.6.0 + )^41,42^ with an *e* value threshold of 1E^−5^. The protein domains were annotated using PfamScan (pfamscan_version)^43^ and InterProScan (v5.35-74.0)^44^ based on InterPro protein databases. The motifs and domains within gene models were identified using PFAM databases^45^. Gene Ontology (GO)^46^ IDs for each gene were obtained from Blast2GO^47^.

### Annotation of noncoding RNA genes

We used transfer RNA (tRNA)scan-SE (v1.3.1)^48^ algorithms with default parameters to identify the genes associated with tRNA. For ribosomal RNA (rRNA) identification, we first downloaded rRNA sequences from closely related species from the Ensembl database. Then, rRNAs in the database were aligned against our genome using BLASTN^41,42^ with a cutoff of *e* value <1e^−5^, identity ≥85% and match length ≥50 bp. microRNA (MiRNAs) and small nuclear RNAs (snRNAs) were identified by Infernal (v1.1.2)^49^ software against the Rfam (v14.1)^45^ database with default parameters.

### Gene family identification

All proteins were extracted and aligned to each other using BLASTP programs (ncbi blast v2.6.0)^42^ with a maximum *e* value of 1e^−5^. To exclude putative fragmented genes, identities with less than 30%, coverage less than 50%, and genes encoding protein sequences that were shorter than 50 bp were filtered out. The OrthoMCL (v14-137)^50^ method was used to cluster genes from these different species into gene families with the parameter “-inflation 1.5.”

### Phylogenetic and gene family analysis

The single-copy orthologous gene protein sequences were aligned with the MUSCLE (v3.8.31)^51^ program, and the corresponding coding DNA sequence alignments were generated and concatenated with the guidance of the protein alignment. RAxML (v8.2.11)^52^ was used to construct the phylogenetic tree with the maximum likelihood method.

Based on the identified gene families and the constructed phylogenetic tree with a predicted divergence time of those species, we used CAFE^53^ to analyze gene family expansion and contraction. This method implemented hypergeometric test algorithms, and the *q* value (false discovery rate (FDR)) was calculated to adjust the *p* value using the R package.

### Synteny analyses

We first performed a whole-genome comparison of the two genomes with the default parameters in the NUCmer tool^54^, filtered the sequence by delta filtering with the −1 parameter, and filtered out collinear fragments with a length less than 10 kb. SNPs and the variations between the two genomes were found using show-snps with the “-rT” parameter and show-diff with the “-rH” parameter, respectively.

### Genomic variations

To explore genetic variations in the *O. fragrans* germplasm, clean reads from the resequencing data of the 122 *Osmanthus* plant accessions were aligned against the OFL genome assembly using Burrows-Wheeler aligner v0.7.17 (BWA)^55^ with default parameters. The 122 accessions were categorized into five groups: the 119 *O. fragrans* accessions formed the ‘Yingui group’, ‘Jingui group’, ‘Dangui group’, and ‘Sijigui group’, and an ‘outgroup’ was formed that included three other *Osmanthus* accessions together with data for *Olea europaea* (Supplementary Table 1).

SNP calling was performed using the Genome Analysis Toolkit v4.1.4.1^56,57^. Briefly, duplicated reads were annotated using MarkDuplicates under default settings. SNPs and indels for each sample were first called using HaplotypeCaller, setting the ploidy to 2 and ERC to GVCF mode. GVCFs were combined using CombineGVCF with the default settings. The final genotyping of the population was performed using GenotypGVCFs under default settings. The SNPs were filtered for quality to apply the following criteria: quality/depth < 2.0 || FS > 60.0 || MQ (quality of the mapped reads of one site) < 40.0 || MQRankSum < −12.5 || ReadPosRankSum < −8.0. The SNPs in the joint genotyping were further filtered to remove SNP sites with MAF < 0.05, sequencing depth < 4, and those that had samples with missing data.

We used Treebest software (v1.9.2) (http://treesoft.sourceforge.net/treebest.shtml) to build an neighbor-joining (NJ) phylogenetic tree with a bootstrap of 100 and visualized the tree using iTOL^58^. GCTA software (v1.91.4 beta3) was used to perform principal component analysis (PCA) with default settings^59^. We also investigated the population structure using ADMIXTURE (v1.3.0), specifying *K* values ranging from two to eight^60^. The most suitable number of ancestral populations was determined by the *K* value with the lowest cross-validation error (CV). PopLDdecay (v3.30) with MaxDist set at 100 was used to calculate the linkage disequilibrium (LD) value of each group^61^.

Population differentiation indices (Fst: Fixation index) between a pair of subpopulations were calculated using VCFtools (v0.1.13)^62^, with a slide window size of 100 kb and step size of 10 kb. To identify regions with differentiation, we took the top 5% regions as candidate regions, from which we performed GO and KEGG enrichment analysis of the genes in these candidate regions.

### Genome-wide association study

GWAS was performed using GAPIT software (v3.0)^63^ based on an mixed linear model and ten principal components as covariates. The SNP association *p* value was adjusted for the FDR using the Benjamini and Hochberg method^64^. The *p* value −log_10(p)_ ≥ 7 was used as a significance threshold. The candidate region was selected as 10 kb upstream and downstream from a significantly associated SNP. Overlapping candidate regions were merged. The results of the GWAS are presented in the form of Manhattan and Q-Q plots. Genes in candidate regions were analyzed based on GO and KEGG enrichment.

### Expression analysis of candidate genes

A qRT-PCR Applied Biosystems 7500 sequence detection system (ABI7500; Thermo Fisher Scientific, Inc.) was used to analyze samples from different tissue parts (root, stem, and leaf) and flowering stages (S1-S6: Bud stage, initial flowering stage, early full flowering stage, full flowering stage, late full flowering stage, abscission stage) of OFL (Supplementary Fig. 1). The qRT-PCR primers were designed using Prime Premier 5 (Supplementary Table 2). The qRT-PCR solution was composed of 2 μL of cDNA, 0.8 μL of each forward and reverse primer, 10 μL of SYBR Mix and 6.4 μL of double-distilled water in a total volume of 15 μL. *Actin’*s expression level was used as a reference, and qRT-PCR amplification was performed using the following conditions: 94 °C for 30 s and 40 cycles of 94 °C for 10 s and 60 °C for 30 s. Relative expression levels were calculated using the 2^–ΔΔCT^ method, and each analysis included three replicates. Significant differences were obtained using SPSS with Duncan’s test at *p* < 0.05.

## Results

### Genome sequencing and assembly

The genome size was estimated by flow cytometry using the method of Dolezel^65^ on a Sysmex CyFlow Ploidy Analyzer (Sysmex Medical Electronics Shanghai Co., Ltd.). The results suggested that the genome size of OFL was ~690 M by referencing *Solanum lycopersicum* and 770 M by referencing *S. tuberosum* (Supplementary Fig. 2). OFL was sequenced to obtain 71 Gb of clean sequence data using the Illumina platform and 174.53 Gb using the PacBio sequencing platform. We generated the 17-mer occurrence distribution using the Illumina data and estimated the genome size to be ~783.63 Mb. The proportion of repeat sequences and the genome’s heterozygosity rate were determined to be ~54.37% and 1.17%, respectively. A 733 Mb genome was assembled using PacBio data containing 575 contigs with a contig N50 of 2.36 Mb, which accounted for 93.54% of the genome size. The contigs were anchored to 23 pseudochromosomes using Hi-C libraries with lengths from 21.89 Mb to 47.60 Mb that anchored 92.41% of the assembled sequences. The final corrected chromosome-level genome was 677 Mb in size, with 541 contigs. The assembled genome was highly complete, with 96.7% of Benchmarking sets of Universal Single Copy Orthologs (BUSCOs) (Table 1). To examine assembly integrity, the continuous long read subreads were realigned onto the final assembly using minimap2 (v2.5) with the default parameters. A total of 99.52% of raw reads could be mapped. Overall, 96.7% complete and 1.0% partial of BUSCOs were identified in the assembled genome, indicating a high completion level. To evaluate the accuracy of the genome at the nucleotide level, Illumina short reads were aligned to the assembly, and we identified 0.0032% homozygous SNPs, indicating a highly accurate genome.

**Table 1:** Comparison of genome sequencing, assembly and annotations of *O. fragrans* ‘Liuyejingui’ (OFL), and the published *O. fragrans* ‘Rixianggui’ (OFR)

Content	OFL genome	OFR genome
Genome size	733.26 Mb	740.71 Mb
Contig number	575	774
Contig N50	2.36 Mb	1.6 Mb
Number of superscaffold chromosomes	23	23
Assembled superscaffold chromosomes size	677.64 Mb	739.37 Mb
Asembled superscaffold chromosomes contigs	541	–
Assembled BUSCOs	96.70%	96.10%
Heterozygosity	1.17%	1.45%
Number of genes	41,252	45,542
Average gene length	5639.29 bp	4065.24 bp
BUSCOs in annotation	96.80%	94.50%

### Genome annotations

We used homology-based and de novo approaches to identify TEs. Our assembly indicated that 447.7 Mb (61.06%) of the assembled genome consisted of repeated regions. Among them, LTR retrotransposons were the most abundant annotations, making up 47.78% of the genome.

We masked repeated regions and proceeded to annotate the genome using a comprehensive strategy including ab initio gene prediction, homology-based gene prediction, and RNA-seq-aided gene prediction. In total, 41,252 protein-coding genes with an average length of 5639 bp were predicted in the assembled OFL genome (Table 1). Approximately 39,068 (~94.71%) of the predicted protein-coding genes of OFL were functionally annotated with known genes, conserved domains, and GO terms. In addition, we identified 148 miRNA, 714 tRNA, 500 rRNA, and 248 snRNA sequences.

We used BUSCO to evaluate the quality of our gene annotation and found that 1562 (96.8%) highly conserved core proteins in embryophyta_odb10 were present in our gene annotation.

### Comparative genomics

**Fig. 1:**
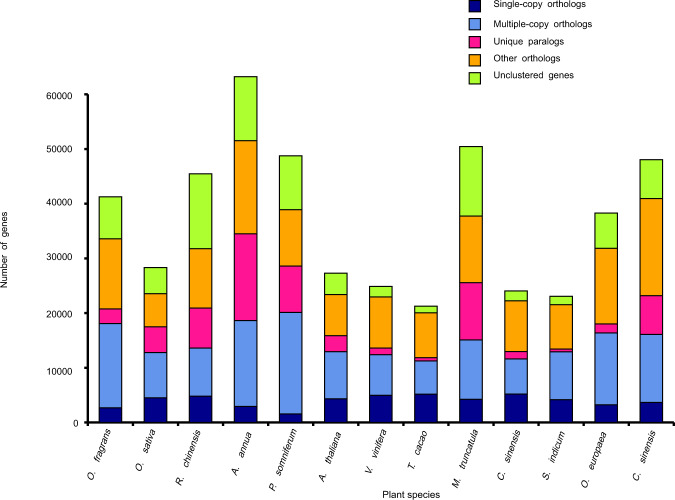
Evolution of gene numbers. The number of genes in various plant species

To investigate the evolution of OFL, we compared its genome to those of other flowering plant species, including *Oryza sativa*, *Papaver somniferum*, *Citrus sinensis*, *Arabidopsis thaliana*, *Theobroma cacao*, *Rosa chinensis*, *Medicago truncatula*, *Vitis vinifera*, *Camellia sinensis*, *Artemisia annua*, *O. europaea*, and *Sesamum indicum*. As a result, we clustered 41,252 genes into 16,107 gene families. A total of 191 genes were identified as shared single-copy orthologous genes (Fig. 1).

**Fig. 2:**
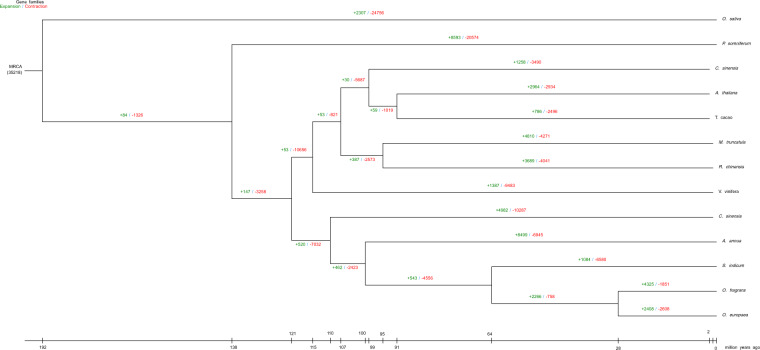
Gene family expansion and contraction analysis of most recent common ancestor in various plant species. The green number represents the number of expanding gene families and the red number represents the number of contracting gene families

To reveal the phylogenetic relationships among OFL and other related species, protein sequences from the 186 filtered single-copy orthologous genes were used for phylogenetic tree reconstruction. The phylogenetic relationship of the other related species was consistent with that in the previous studies^13^. According to the divergence times and phylogenetic relationships, 4325 gene families were significantly expanded in the OFL genome, and 1851 gene families were significantly contracted (*p* < 0.05). Those expanded gene families included 3274 significantly enriched (*q* value < 0.05) KEGG pathways (Fig. 2). Genes involved in the biosynthesis of monoterpenoids, diterpenoids, sesquiterpenoids, triterpenoids, limonene, and carotenoids were expanded.

### Genomic variations and population evolution

The evolutionary relationships of varieties of *O. fragrans* with different flower colors are still unclear. Thus, we collected 119 phenotypically diverse populations of *O. fragrans* cultivars and their close relatives for whole-genome resequencing to investigate the genetic architecture of floral color traits using the OFL genome as a reference genome. Each plant was subjected to whole-genome sequencing on the MGI-SEQ 2000 platform, obtaining on average 11 G of data per plant, which is approximately 15X coverage based on the genome size estimates. We mapped the sequencing data to the OFL genome with, on average, a 98% mapping rate. Approximately 86.7% of the genome was covered by at least four reads, and 68.6% of the genome was covered by at least ten reads. We performed SNP calling based on the mapping data and identified 11.44 million SNPs per plant on average. After filtering SNP positions with sequencing depth <4, MAF < 0.05 and a requirement of no missing data, we obtained a total of 2,072,100 SNPs.

**Fig. 3:**
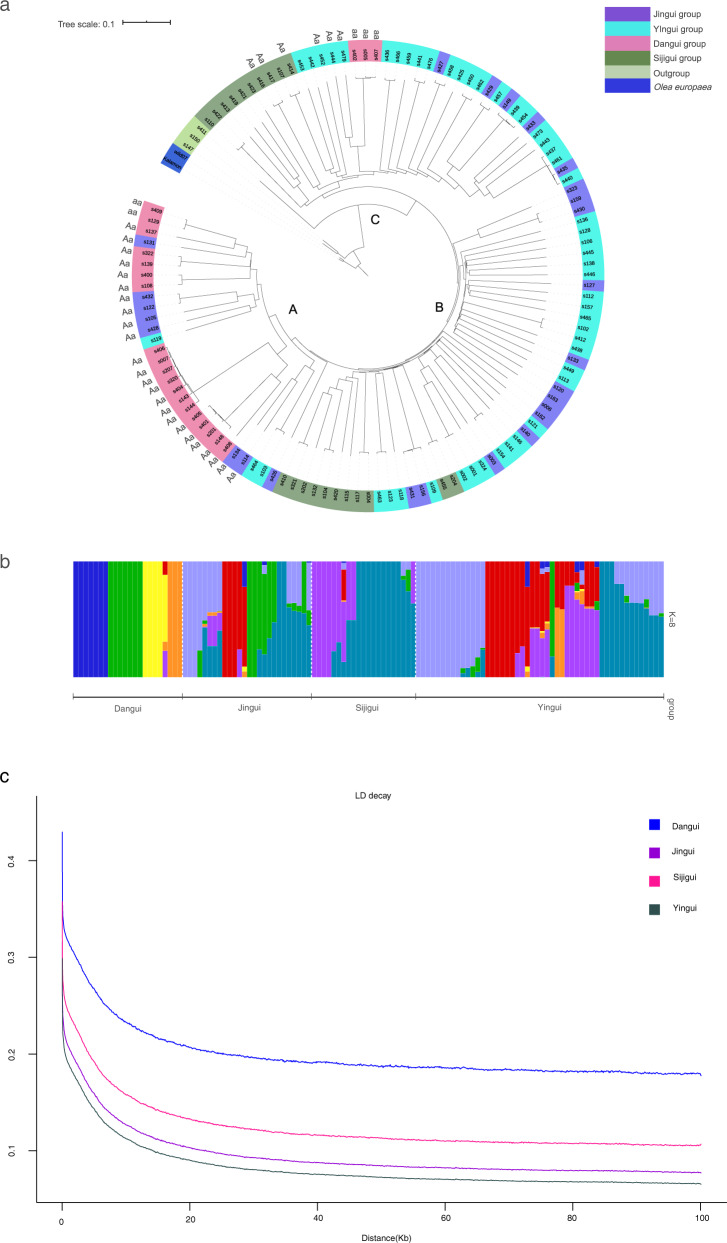
Genomic variations and population evolution analysis. **a** Phylogenetic tree of 122 *Osmanthus* accessions and 2 *Olea europaea*; **b** population structure analysis; **c** LD plot

We constructed a phylogenetic tree using the NJ method. We found three general clusters: cluster A, which consisted mainly of the ‘Dangui group’, and clusters B and C, which consisted predominately of separate subgroups from the ‘Yingui group’ and ‘Sijigui group’. The ‘Jingui group’ was found to be more dispersed among each of these three tree clusters. From our PCA, we observed similar outcomes, with the ‘Dangui group’ plants being more separated from other *Osmanthus* varieties. The ‘Yingui group’ was separated into two subgroups, both of which clustered more closely with the ‘Sijigui group’ (Fig. 3a).

The population structure analysis for these populations showed that when the cluster (k) was 8, the least CV error was detected. The “Dangui group” showed the lowest amount of mixture, while the other varieties showed some common ancestry from different *Osmanthus* ancestors (Fig. 3b). The LD decay plots for the four populations showed that the “Dangui group” flattened out the fastest, followed by the “Sijigui group”, the “Jingui group”, and finally the “Yingui group” (Fig. 3c).

### GWAS analysis of ornamental traits

We examined the important ornamental traits in *Osmanthus* varieties to look for markers that are significantly associated with petal color.

**Fig. 4:**
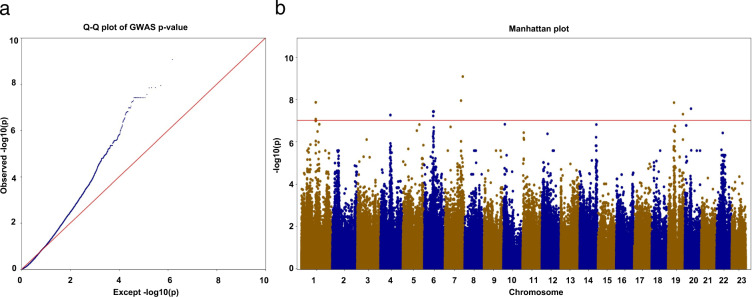
Genome-wide association analysis for imputed SNPs interaction with petal color. (**a**) Q–Q plot; (**b**) Manhattan plot

*Osmanthus* flower colors are categorized as white, yellow, and orange/red. A total of 22 plants were categorized as having orange/red flowers. The CMLM model identified 25 significant loci containing 35 genes (Fig. 4). The significant candidate regions were distributed on six chromosomes. The identified candidate genes included cytochrome c oxidase (LYG001209), protein transport protein sec16 (SEC16B, LYG008575), ethylene-responsive transcription factor 2 (ERF2, LYG012560), cyclin-dependent kinase D-1 (LYG012568), auxin response factor 11 (ARF11, LYG014851), E3 ubiquitin-protein ligase (Mib, LYG032877), and 9-cis-epoxycarotenoid dioxygenase (NCED6, LYG034219).

To further filter genes that may contribute to orange/red flower color in *O. fragrans* we examined the Fst values between the ‘Dangui group’ and the other three groups. Of the 35 genes, 24 genes were within the top 5% of Fst values between the ‘Dangui group’ and the other groups; these genes included ERF2 (LYG012560), two-component response regulator-like APRR2 (APRR2, LYG012584), phosphomevalonate kinase (PMVK, LYG012595), ARF11 (LYG014851), and NCED6 (LYG034219) (Table 2).

**Table 2:** SNPs associated with petal color

Num.	Gene	SNP position	SNP *p* value	FDR	Annotation
1	LYG012558	6:13817422	3.74E^−08^	0.00146611	RGG repeats nuclear RNA binding protein A
2	LYG012559	6:13819023	3.74E^−08^	0.00146611	Phospholipase A1-Ibeta2, chloroplastic
3	LYG012560				Ethylene-responsive transcription factor 2
4	LYG012563	6:13918308	6.10E^−08^	0.001891581	uncharacterized
5	LYG012568	6:14012303	3.74E^−08^	0.00146611	Cyclin-dependent kinase D-1
6	LYG012579	6:14272320	3.74E^−08^	0.00146611	Lon protease homolog 2, peroxisomal
7	LYG012580				Protein phosphatase 2C
8	LYG012581	6:14293904	3.74E^−08^	0.00146611	Pentatricopeptide repeat-containing protein
9	LYG012582				Type I inositol polyphosphate 5-phosphatase 2
10	LYG012584	6:14339148	3.74E^−08^	0.00146611	Two-component response regulator-like APRR2
11	LYG012585	6:14381541	3.74E^−08^	0.00146611	Haloacid dehalogenase-like hydrolase domain-containing protein
12	LYG012586	6:14384154	3.74E^−08^	0.00146611	Regulator of nonsense transcripts 1
13	LYG012587	6:14392645	3.74E^−08^	0.00146611	AT-hook motif nuclear-localized protein 10
14	LYG012588	6:14413335	3.74E^−08^	0.00146611	uncharacterized
15	LYG012589				Protein CLMP1
16	LYG012595	6:14571827	3.74E^−08^	0.00146611	Phosphomevalonate kinase, peroxisomal
17	LYG012596				Extra-large guanine nucleotide-binding protein 3
18	LYG014851	7:25078341	1.13E^−08^	0.00146611	Auxin response factor 11
19	LYG014852	7:27860295	8.32E^−10^	0.000618978	uncharacterized
20	LYG014853				L-ascorbate oxidase
21	LYG033390	19:22751430	4.99E^−08^	0.001771043	uncharacterized
22	LYG033391				uncharacterized
23	LYG033392				uncharacterized
24	LYG034219	20:9512838	2.72E^−08^	0.00146611	9-cis-epoxycarotenoid dioxygenase NCED6, chloroplastic

**Fig. 5:**
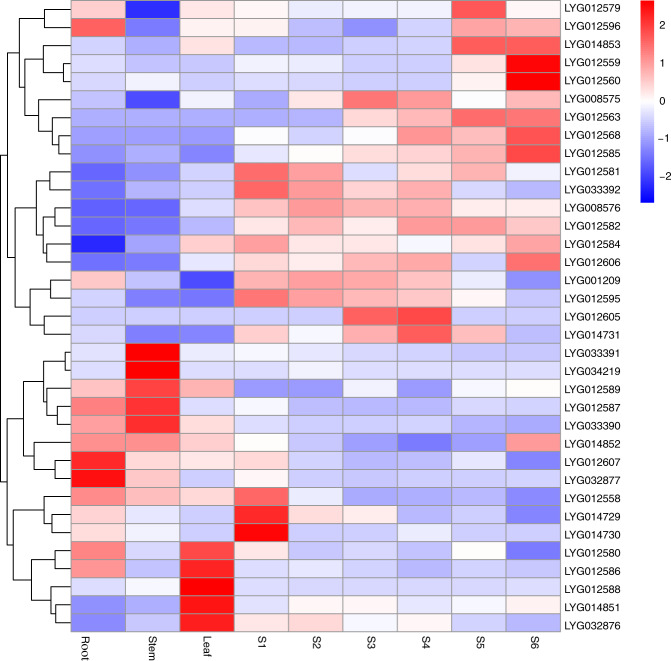
The expression pattern of genes associated with petal color. Heatmap showing the expression of 35 genes identified by the CMLM model in different tissue parts and flowering stages

To validate the differential expression of candidate genes significantly associated with flower color-related phenotypes in *O. fragrans*, we performed RNA-seq analysis by sequencing nine transcriptomes of OFL (roots, stems, leaves, and flowers for six different flowering stages, with three biological replicates per sample). The results showed that of these 35 genes, 12 were differentially expressed during flowering (Fig. 5).

### Variations in *CCD4* gene loci

**Fig. 6:**
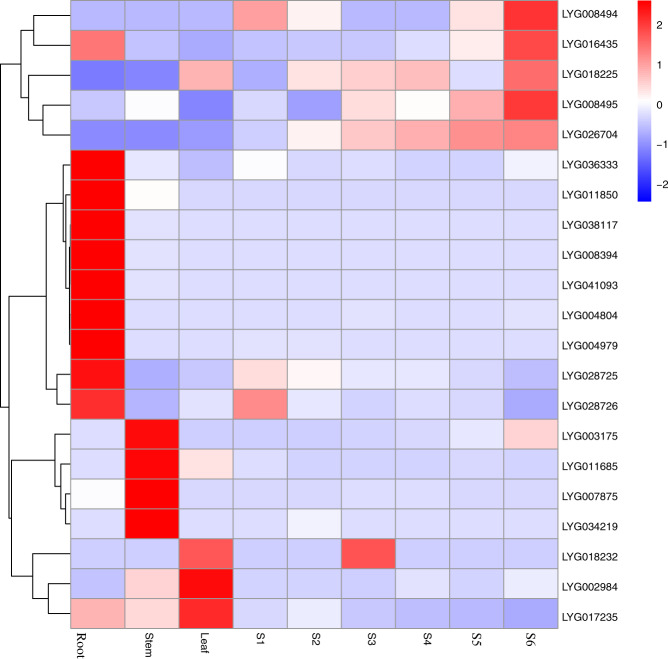
The expression pattern of *CCD* gene family in *O. fragrans*. Heatmap showing the expression of *CCD* genes in different tissue parts and flowering stages

It was reported that the orange/red color of *Osmanthus* varieties in the ‘Dangui group’ was due to the accumulation of carotenoids^10^. We then analyzed the expression pattern of the *CCD* gene family in different tissue parts (root, stem, and leaf) and flowering stages (S1-S6) of OFL. There were a total of four *CCD4* genes in the OFL genome, including *CCD4a* (LYG004804) located on chromosome 2, *CCD4b* (LYG008494) and *CCD4c* (LYG008495) located on chromosome 4, and *CCD4d* (LYG026704) located on chromosome 15. The results showed that the *CCD4b, CCD4c*, and *CCD4d g*enes were differentially expressed during the flowering process and that the *CCD4a* gene was expressed at a high level only in the root (Fig. 6).

**Fig. 7:**
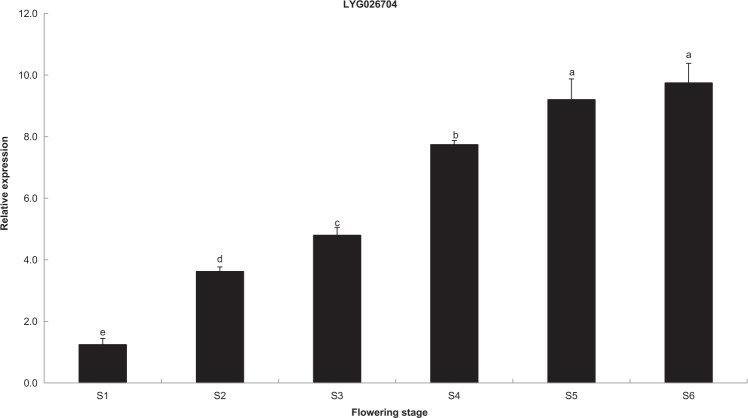
Transcript levels of the *CCD4d* gene in different flowering stages. Real-time PCR analysis of *CCD4d* genes in different flowering stages and the data represent the means ± SD of the three replicates from three independent experiments with significant differences at *P* = 0.05 level

Real-time PCR analysis was then carried out to screen for *CCD4* members that may contribute to orange/red petal color. The results showed that *CCD4b* and *CCD4c*, located on chromosome 4, had abnormal gene structures and thus could not be cloned. The only functional member of *CCD4* that was differentially expressed during flowering was *CCD4d*, which was the same one identified in previous studies (Fig. 7)^8,9,12^.

**Fig. 8:**
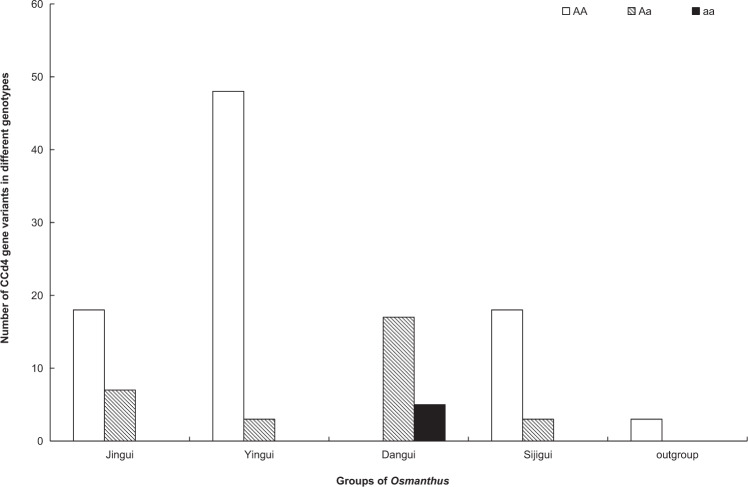
The 34-bp deletion analysis of *CCD4d* gene. Numbers of *CCD4d* gene variants in different genotypes

Surprisingly, we found an allele with a 34-bp deletion in the first coding region of the *CCD4d* gene. The 34-bp deletion allele, denoted by ‘a’ (the wild allele is denoted by ‘A’), existed in all varieties of the ‘Dangui group’. We analyzed 122 resequenced samples, and the results showed that none of the genotype AA samples were from varieties in the ‘Dangui group’, that all genotype aa samples were varieties in the ‘Dangui group’, and that genotype Aa samples included varieties from all four groups, including those with white, yellow, and orange/red flower colors (Fig. 8, Supplementary Table 3). More interestingly, these Aa genotype samples in other groups were clustered closely to the ‘Dangui group’ on the phylogenetic tree (Fig. 3a). These results showed that the frameshift mutation of the *CCD4* gene is probably related to the formation of orange/red flower color in *O. fragrans*.

## Discussion

As most cultivars of *O. fragrans* bloom in autumn, we chose two cultivars of *O. fragrans*, ‘Liuyejingui’ in the Jingui group and *O. fragrans* ‘Gecheng Dangui’ in the Dangui group, as plant materials for the preliminary experiment. The results showed that *O. fragrans* ‘Gecheng Dangui’ had a greater heterozygosity, 1.35%, than ‘Liuyejingui’ (unpublished observation). Here, we present a genome for OFL, which is a typical autumn-flowering cultivar used for economic harvest and compare it with the published OFR genome^13^. Compared with that of the published genome, the size of the ‘Liuyejingui’ genome is similar; however, our contig N50 is ~2.36 Mb, which is much larger, and our assembled and annotated BUSCO evaluation results are better (Table 1). The average length of our OFL genome gene is ~5.6 kb, which is higher than that in the published results, indicating that our gene structure annotation is more complete. Moreover, the calculated level of heterozygosity was 1.17% in *O. fragrans* ‘Liuyejinggui’, while it was higher (1.45%) in OFR (Table 1). Synteny analyses were then performed, and the results showed a high collinearity between these two assemblies, except for some structural variation (Supplementary Fig. 3). In summary, we constructed a high-quality reference genome.

As an important phenotypic trait, *O. fragrans* flower color has always been an essential basis for its classification and evolution^66^. Compared with the traditional morphological classification of the four groups of *O. fragrans*, the results of the population structure analysis show that the varieties are clustered into three groups. These results suggest that in the long-term evolutionary process, under the effects of natural and artificial selection, these varieties’ genetic material has developed apparent regional clustering, which implies the different origins of *O. fragrans* in the southeastern and southwestern regions of China.^1,2^ This subpopulation differentiation caused by geographical isolation has also been reported in other species^67–69^.

It has been reported that the ‘Sijigui group’ is relatively close to the wild progenitor and that the ‘Yingui group’ is the more original of the three autumn-flowering groups; the ‘Jingui group’ appears later, and the ‘Dangui group’ appears latest^1,2,66^. The white/yellowish-white flower color is likely the original character, according to the flower color analysis of wild species in *Osmanthus*. In contrast, the yellow to orange-red flower colors were not found in the wild species. They appear only under certain cultivation conditions, which indicates an evolutionary trait in the process of breed evolution.^1^ The results of LD decay analysis are consistent with the conclusions of traditional osmanthus resource surveys, with the exception of the ‘Sijigui group’, which suggests that varieties in the ‘Sijigui group’ are probably artificially domesticated species rather than wild species. DNA barcoding with the *trnS-G* and *nad7* introns of 2 *O. fragrans* groups showed similar results: Sijigui and Dangui clustered together^70^.

In addition, compared with the other three groups of *O. fragrans*, most of the varieties in the ‘Dangui group’ eliminated their regional aggregation and clustered independently, indicating that the ‘Dangui group’ was probably a bud sport that appeared in a particular area in the past. Under long-term artificial directional selection, a stable group of varieties was formed and then introduced and cultivated elsewhere. It has been suggested that the color of *O. fragrans* was described only as “white” in or before the Tang Dynasty in ancient Chinese texts but as both “white” and “yellow” during the Song Dynasty; the description of the red/orange color of *O. fragrans* appears only in the late Song Dynasty, which provides some support for our inference^1,2^.

To further explore the origin and evolution of flower color in *O. fragrans*, we identified significant QTLs and genomic regions associated with red/orange color through a GWAS in which several genes, such as PMVK, ERF2, and APRR2, were characterized. Among them, APRR2 has been reported to support carotenoid biofortification; it also increases the plastid number and area as well as pigment content, enhancing the levels of chlorophyll in immature unripe fruits and carotenoids in red ripe fruits when it is overexpressed^71,72^. ERF6 was reported to bind to the *CCD4* promoter and stimulate *CCD4* expression, thereby regulating the synthesis of β-ionone in *O. fragrans* petals^9^.

The differences in the flower color of *O. fragrans* varieties are attributable mainly to the level of carotenoids in the flowers^10^. Moreover, *CCD1* and *CCD4* are crucial contributors to the cleavage of α-carotene and β-carotene^11,12^. The study of ‘Redhaven’ peach and its white-fleshed mutant showed that *CCD1*s contribute only to volatile production, while *CCD4*s are likely to control carotenoid degradation^73^. In *O. fragrans*, the most crucial factor determining the diversity of carotenoid concentrations was also the differential expression level of *CCD4*^9,10^. In the present study, we found a surprising 34-bp deletion in the first coding region of the *CCD4d* gene in all varieties of the “Dangui group”, and this frameshift mutation existed in at least one site in both alleles. This result suggests that the orange/red color of the ‘Dangui group’ is probably related to the *CCD4d* mutation. Variations in *CCD4* gene loci contribute to differences in carotenoid and apocarotenoid content among varieties of the same species and have also been found in citrus and petunia^74,75^. On the other hand, the Aa genotype results occurring in the Jingui, Yingui, Dangui, and Sijigui phenotypes also indicated that the *CCD4d* gene is probably not the only major gene that controls the biological metaboli*s*m of carotenoids. In addition to the *CCD4d* gene, there are likely other regulatory factors, such as ERF2 and APRR2, that were determined by GWAS to regulate the metabolism of carotenoids. Further studies should endeavor to study these candidate genes involved in flower color formation in order to elucidate the mechanism of the formation of orange/red color in *O. fragrans* flowers.

## Conclusion

In this study, we successfully sequenced and assembled a reference genome for OFL, an autumn-flowering cultivar harvested for its economic value, by combining results from the Illumina, PacBio and Hi-C platforms. We also reported on genomic variations and population evolution by resequencing 119 *Osmanthus* accessions from four groups of *O. fragrans* to explore the origin and evolution of flower color. Significant QTLs and genomic regions were identified in which several genes that were positively associated with petal color, such as ERF2 and APRR2, were located. On the other hand, the frameshift mutation of the *CCD4* gene is probably related to the formation of orange/red flower color in *O. fragrans*. The reference genome sequence and genomic variation map of *O. fragrans* provide insights into the genome evolution of the *O. fragrans* species, benefiting both basic and applied plant biologists.

## Supplementary information

Supplementary File-Marked Up

## Data Availability

Raw sequencing reads of all *Osmanthus* plant accessions reported in this study have been deposited into the public database of the National Center of Biotechnology Information (NCBI) BioProject under the accession number PRJNA679852. RNA-seq raw data were also deposited under these NCBI accessions.
