# Supplementary material for: Whole-genome resequencing of Osmanthus fragrans provides insights into flower color evolution
Source: Hortic Res. 2021 May 1;8:98. doi: 10.1038/s41438-021-00531-0 (PMC8087690; doi:10.1038/s41438-021-00531-0)
Supplement: Supplementary file 1 — Supplementary File-Marked Up [file 41438_2021_531_MOESM1_ESM.doc]

**Supplementary Table 1 Information of 122 samples**

| **Number** | **Samples** | **Group** | **Cultivar** | **Sampling Place** | **Introduction Place** |
| --- | --- | --- | --- | --- | --- |
| **1** | **s005** | Dangui group | Lianzidangui | Wuhan (114°21’W, 30°29’N) | Sichuan Province |
| **2** | **s007** | Dangui group | Dangui | Wuhan (N30°55′E114°38′) | Hubei Province |
| **3** | **s108** | Dangui group | Puchengdangui | Xianning (N29°86′E114°37′) | Zhejiang Province |
| **4** | **s129** | Dangui group | Zhuangyuanhong | Xianning (N29°86′E114°37′) | Zhejiang Province |
| **5** | **s137** | Dangui group | Zuijihong | Xianning (N29°86′E114°37′) | Zhejiang Province |
| **6** | **s139** | Dangui group | Yingyedangui | Xianning (N29°86′E114°37′) | Zhejiang Province |
| **7** | **s143** | Dangui group | Pingmaihong | Xianning (N29°49′E114°19′) | Zhejiang Province |
| **8** | **s144** | Dangui group | Mantiaohong | Xianning (N29°49′E114°19′) | Hubei Province |
| **9** | **s148** | Dangui group | Sushengdangui | Xianning (N29°49′E114°19′) | Zhejiang Province |
| **10** | **s201** | Dangui group | Xionghuanggui | Wuhan (N30°55′E114°38′) | Sichuan Province |
| **11** | **s207** | Dangui group | Dayezhushagui | Wuhan (N30°55′E114°38′) | Sichuan Province |
| **12** | **s320** | Dangui group | Zhushagui | Xianning (N29°51′E114°19′) | Hubei Province |
| **13** | **s322** | Dangui group | Xiaoyedangui | Xianning (N29°51′E114°19′) | Hubei Province |
| **14** | **s400** | Dangui group | Fujianhong | Wuhan (N30°55′E114°38′) | Zhejiang Province |
| **15** | **s401** | Dangui group | Chenghongdangui | Wuhan (N30°55′E114°38′) | Sichuan Province |
| **16** | **s402** | Dangui group | Lianzidangui | Wuhan (N30°55′E114°38′) | Sichuan Province |
| **17** | **s404** | Dangui group | Zhushadangui | Wuhan (N30°55′E114°38′) | Sichuan Province |
| **18** | **s405** | Dangui group | Zaohongdangui | Wuhan (N30°55′E114°38′) | Sichuan Province |
| **19** | **s406** | Dangui group | Xiaoyezhushagui | Wuhan (N30°55′E114°38′) | Sichuan Province |
| **20** | **s407** | Dangui group | Zaohuazidangui | Wuhan (N30°55′E114°38′) | Sichuan Province |
| **21** | **s408** | Dangui group | Yanhongui | Wuhan (N30°55′E114°38′) | Sichuan Province |
| **22** | **s409** | Dangui group | Xiangshandangui | Wuhan (N30°55′E114°38′) | Zhejiang Province |
| **23** | **s003** | Jingui group | Jingui | Xianning (N29°43′E114°18′) | Hubei Province |
| **24** | **s006** | Jingui group | Liuyejingui | Wuhan (N30°55′E114°38′) | Hubei Province |
| **25** | **s105** | Jingui group | Xiaoyan | Xianning (N29°86′E114°37′) | Zhejiang Province |
| **26** | **s114** | Jingui group | Dahuajingui | Xianning (N29°86′E114°37′) | Zhejiang Province |
| **27** | **s120** | Jingui group | Kuoyejingui | Xianning (N29°86′E114°37′) | Zhejiang Province |
| **28** | **s122** | Jingui group | Jinqiugui | Xianning (N29°86′E114°37′) | Zhejiang Province |
| **29** | **s127** | Jingui group | Chunjin | Xianning (N29°86′E114°37′) | Zhejiang Province |
| **30** | **s131** | Jingui group | Xiaoyezijin | Xianning (N29°86′E114°37′) | Zhejiang Province |
| **31** | **s133** | Jingui group | Chuizhijingui | Xianning (N29°86′E114°37′) | Zhejiang Province |
| **32** | **s134** | Jingui group | Yichuanjin | Xianning (N29°86′E114°37′) | Zhejiang Province |
| **33** | **s140** | Jingui group | Chibaohuang | Xianning (N29°49′E114°19′) | Hubei Province |
| **34** | **s149** | Jingui group | Sushengjingui | Xianning (N29°49′E114°19′) | Hubei Province |
| **35** | **s156** | Jingui group | Hangzhouhuang | Xianning (N29°49′E114°19′) | Zhejiang Province |
| **36** | **s159** | Jingui group | Qinzhanbizhu | Xianning (N29°49′E114°19′) | Hubei Province |
| **37** | **s162** | Jingui group | Yangguang | Xianning (N29°49′E114°19′) | Hubei Province |
| **38** | **s163** | Jingui group | Xianningshangui | Xianning (N29°49′E114°19′) | Hubei Province |
| **39** | **s323** | Jingui group | Jiezijingui | Xianning (N29°51′E114°19′) | Hubei Province |
| **40** | **s426** | Jingui group | Dahuajingui | Wuhan (N30°55′E114°38′) | Sichuan Province |
| **41** | **s427** | Jingui group | Zijingui | Wuhan (N30°55′E114°38′) | Sichuan Province |
| **42** | **s428** | Jingui group | Wandianjin | Wuhan (N30°55′E114°38′) | Zhejiang Province |
| **43** | **s429** | Jingui group | Jinqiuzao | Wuhan (N30°55′E114°38′) | Sichuan Province |
| **44** | **s430** | Jingui group | Huangchuanjingui | Wuhan (N30°55′E114°38′) | Zhejiang Province |
| **45** | **s431** | Jingui group | Qiugui | Wuhan (N30°55′E114°38′) | Zhejiang Province |
| **46** | **s432** | Jingui group | Jinqiugui | Wuhan (N30°55′E114°38′) | Zhejiang Province |
| **47** | **s433** | Jingui group | Ruanyejingui | Wuhan (N30°55′E114°38′) | Sichuan Province |
| **48** | **s435** | Jingui group | Chenghuangjingui | Wuhan (N30°55′E114°38′) | Sichuan Province |
| **49** | **s004** | Sijigui group | Tianxiangtaige | Wuhan (N30°55′E114°38′) | Zhejiang Province |
| **50** | **s104** | Sijigui group | Xiaoyesijigui | Xianning (N29°86′E114°37′) | Zhejiang Province |
| **51** | **s107** | Sijigui group | Dongxianghong | Xianning (N29°86′E114°37′) | Zhejiang Province |
| **52** | **s110** | Sijigui group | Fodingzhu | Xianning (N29°86′E114°37′) | Zhejiang Province |
| **53** | **s115** | Sijigui group | Ziyue | Xianning (N29°86′E114°37′) | Zhejiang Province |
| **54** | **s117** | Sijigui group | Tianxiangtaige | Xianning (N29°86′E114°37′) | Zhejiang Province |
| **55** | **s132** | Sijigui group | Yuanyesijigui | Xianning (N29°86′E114°37′) | Zhejiang Province |
| **56** | **s202** | Sijigui group | Tanzhuang | Wuhan (N30°55′E114°38′) | Zhejiang Province |
| **57** | **s204** | Sijigui group | Dayesijigui | Wuhan (N30°55′E114°38′) | Sichuan Province |
| **58** | **s321** | Sijigui group | Sijigui | Xianning (N29°51′E114°19′) | Zhejiang Province |
| **59** | **s410** | Sijigui group | Sijigui | Wuhan (N30°55′E114°38′) | Zhejiang Province |
| **60** | **s413** | Sijigui group | Xiaoronghuang | Wuhan (N30°55′E114°38′) | Sichuan Province |
| **61** | **s414** | Sijigui group | Yueyuehong | Wuhan (N30°55′E114°38′) | Sichuan Province |
| **62** | **s416** | Sijigui group | Chenghuangsijigui | Wuhan (N30°55′E114°38′) | Sichuan Province |
| **63** | **s417** | Sijigui group | Fuzhousijigui | Wuhan (N30°55′E114°38′) | Sichuan Province |
| **64** | **s419** | Sijigui group | Guizisijigui | Wuhan (N30°55′E114°38′) | Sichuan Province |
| **65** | **s420** | Sijigui group | Tiannvsanhua | Wuhan (N30°55′E114°38′) | Zhejiang Province |
| **66** | **s421** | Sijigui group | Juyedijigui | Wuhan (N30°55′E114°38′) | Sichuan Province |
| **67** | **s422** | Sijigui group | Xiaoyefodingzhu | Wuhan (N30°55′E114°38′) | Sichuan Province |
| **68** | **s423** | Sijigui group | Rixianggui | Wuhan (N30°55′E114°38′) | Sichuan Province |
| **69** | **s455** | Sijigui group | Chiyesijigui | Wuhan (N30°55′E114°38′) | Zhejiang Province |
| **70** | **s001** | Yingui group | Boyeyingui | Xianning (N29°44′E114°19′) | Hubei Province |
| **71** | **s002** | Yingui group | Boyeyingui | Xianning (N29°51′E114°19′) | Hubei Province |
| **72** | **s102** | Yingui group | Yinhui | Xianning (N29°86′E114°37′) | Zhejiang Province |
| **73** | **s103** | Yingui group | Suyu | Xianning (N29°86′E114°37′) | Zhejiang Province |
| **74** | **s106** | Yingui group | Yangmeiyeyingui | Xianning (N29°86′E114°37′) | Zhejiang Province |
| **75** | **s109** | Yingui group | Zaohuang | Xianning (N29°86′E114°37′) | Zhejiang Province |
| **76** | **s112** | Yingui group | Qingjian | Xianning (N29°86′E114°37′) | Zhejiang Province |
| **77** | **s113** | Yingui group | Ehuang | Xianning (N29°86′E114°37′) | Zhejiang Province |
| **78** | **s118** | Yingui group | Changyebizhu | Xianning (N29°86′E114°37′) | Zhejiang Province |
| **79** | **s119** | Yingui group | Yulinglong | Xianning (N29°86′E114°37′) | Zhejiang Province |
| **80** | **s121** | Yingui group | Baijie | Xianning (N29°86′E114°37′) | Zhejiang Province |
| **81** | **s123** | Yingui group | Yinsu | Xianning (N29°86′E114°37′) | Zhejiang Province |
| **82** | **s128** | Yingui group | Juanyehuang | Xianning (N29°86′E114°37′) | Zhejiang Province |
| **83** | **s136** | Yingui group | Yingzhiyingui | Xianning (N29°86′E114°37′) | Zhejiang Province |
| **84** | **s138** | Yingui group | Zhaiyeyingui | Xianning (N29°86′E114°37′) | Zhejiang Province |
| **85** | **s141** | Yingui group | Liuyeyin | Xianning (N29°49′E114°19′) | Hubei Province |
| **86** | **s146** | Yingui group | Yinling | Xianning (N29°49′E114°19′) | Hubei Province |
| **87** | **s154** | Yingui group | Liuyesugui | Xianning (N29°49′E114°19′) | Hubei Province |
| **88** | **s157** | Yingui group | Jiangnanliren | Xianning (N29°49′E114°19′) | Hubei Province |
| **89** | **s324** | Yingui group | Zaoyingui | Xianning (N29°51′E114°19′) | Hubei Province |
| **90** | **s412** | Yingui group | Yinxing | Wuhan (N30°55′E114°38′) | Hubei Province |
| **91** | **s425** | Yingui group | Zhonghuahonglonggui | Wuhan (N30°55′E114°38′) | Sichuan Province |
| **92** | **s436** | Yingui group | Juanbanyingui | Wuhan (N30°55′E114°38′) | Guangxi Province |
| **93** | **s437** | Yingui group | Chengduzaoyingui | Wuhan (N30°55′E114°38′) | Sichuan Province |
| **94** | **s438** | Yingui group | Juanyehuang | Wuhan (N30°55′E114°38′) | Sichuan Province |
| **95** | **s439** | Yingui group | Baijian | Wuhan (N30°55′E114°38′) | Sichuan Province |
| **96** | **s440** | Yingui group | Hongjian | Wuhan (N30°55′E114°38′) | Sichuan Province |
| **97** | **s441** | Yingui group | Yulianyinsi | Wuhan (N30°55′E114°38′) | Sichuan Province |
| **98** | **s442** | Yingui group | Mijieyingui | Wuhan (N30°55′E114°38′) | Sichuan Province |
| **99** | **s443** | Yingui group | Xiaoyewanyingui | Wuhan (N30°55′E114°38′) | Sichuan Province |
| **100** | **s444** | Yingui group | Huangrui | Wuhan (N30°55′E114°38′) | Sichuan Province |
| **101** | **s445** | Yingui group | Wanyin | Wuhan (N30°55′E114°38′) | Sichuan Province |
| **102** | **s446** | Yingui group | Dayewanyingui | Wuhan (N30°55′E114°38′) | Sichuan Province |
| **103** | **s449** | Yingui group | Yuchengzaogui | Wuhan (N30°55′E114°38′) | Sichuan Province |
| **104** | **s450** | Yingui group | Juanyeyingui | Wuhan (N30°55′E114°38′) | Sichuan Province |
| **105** | **s452** | Yingui group | Danlingxiangyun | Wuhan (N30°55′E114°38′) | Sichuan Province |
| **106** | **s453** | Yingui group | Yuzhu | Wuhan (N30°55′E114°38′) | Sichuan Province |
| **107** | **s454** | Yingui group | Yinzhanbizhu | Wuhan (N30°55′E114°38′) | Sichuan Province |
| **108** | **s457** | Yingui group | Houyechiyingui | Wuhan (N30°55′E114°38′) | Sichuan Province |
| **109** | **s458** | Yingui group | Pingyeyingui | Wuhan (N30°55′E114°38′) | Guangxi Province |
| **110** | **s459** | Yingui group | Xiziyingui | Wuhan (N30°55′E114°38′) | Sichuan Province |
| **111** | **s461** | Yingui group | Xiaohuayingui | Wuhan (N30°55′E114°38′) | Guangxi Province |
| **112** | **s462** | Yingui group | Furonggui | Wuhan (N30°55′E114°38′) | Sichuan Province |
| **113** | **s463** | Yingui group | Suiyin | Wuhan (N30°55′E114°38′) | Zhejiang Province |
| **114** | **s464** | Yingui group | Suyu | Wuhan (N30°55′E114°38′) | Zhejiang Province |
| **115** | **s465** | Yingui group | Puxinguihua | Wuhan (N30°55′E114°38′) | Sichuan Province |
| **116** | **s466** | Yingui group | Zigeng | Wuhan (N30°55′E114°38′) | Sichuan Province |
| **117** | **s473** | Yingui group | Yingui7hao | Wuhan (N30°55′E114°38′) | Guangxi Province |
| **118** | **s476** | Yingui group | Yulianbizhu | Wuhan (N30°55′E114°38′) | Guangxi Province |
| **119** | **s479** | Yingui group | Yingui4hao | Wuhan (N30°55′E114°38′) | Guangxi Province |
| **120** | **S147** | Out group | *O.heterophyllus*(G. Don) P. S. Green var. *Heterophyllus* | Xianning (N29°49′E114°19′) | Zhejiang Province |
| **121** | **S150** | Out group | *O. cooperi* | Xianning (N29°49′E114°19′) | Zhejiang Province |
| **122** | **S411** | Out group | *O. × fortunei* | Wuhan (N30°55′E114°38′) | Zhejiang Province |

**Supplementary Table 2 Primer information of qRT-PCR**

| **Gene Number** | **Primer** | **Primer sequence (5'to3')** |
| --- | --- | --- |
| **LYG026704** | **F** | CGACGGAACAAAACAACCC |
|  | **R** | CGAGGCGAGGCACTTTTC |

**Supplementary Table 3 The 34-bp deletion allele of 122 samples**

| **Number** | **Samples** | **genotype** |
| --- | --- | --- |
| **1** | **s001** | AA |
| **2** | **s002** | AA |
| **3** | **s003** | AA |
| **4** | **s006** | AA |
| **5** | **s102** | AA |
| **6** | **s103** | AA |
| **7** | **s104** | AA |
| **8** | **s106** | AA |
| **9** | **s107** | AA |
| **10** | **s109** | AA |
| **11** | **s110** | AA |
| **12** | **s112** | AA |
| **13** | **s113** | AA |
| **14** | **s115** | AA |
| **15** | **s117** | AA |
| **16** | **s118** | AA |
| **17** | **s119** | AA |
| **18** | **s120** | AA |
| **19** | **s121** | AA |
| **20** | **s123** | AA |
| **21** | **s127** | AA |
| **22** | **s128** | AA |
| **23** | **s132** | AA |
| **24** | **s133** | AA |
| **25** | **s136** | AA |
| **26** | **s138** | AA |
| **27** | **s140** | AA |
| **28** | **s141** | AA |
| **29** | **s146** | AA |
| **30** | **s147** | AA |
| **31** | **s149** | AA |
| **32** | **s150** | AA |
| **33** | **s154** | AA |
| **34** | **s156** | AA |
| **35** | **s157** | AA |
| **36** | **s159** | AA |
| **37** | **s162** | AA |
| **38** | **s163** | AA |
| **39** | **s202** | AA |
| **40** | **s204** | AA |
| **41** | **s321** | AA |
| **42** | **s323** | AA |
| **43** | **s324** | AA |
| **44** | **s410** | AA |
| **45** | **s411** | AA |
| **46** | **s412** | AA |
| **47** | **s413** | AA |
| **48** | **s419** | AA |
| **49** | **s420** | AA |
| **50** | **s421** | AA |
| **51** | **s422** | AA |
| **52** | **s423** | AA |
| **53** | **s425** | AA |
| **54** | **s426** | AA |
| **55** | **s427** | AA |
| **56** | **s429** | AA |
| **57** | **s430** | AA |
| **58** | **s431** | AA |
| **59** | **s433** | AA |
| **60** | **s435** | AA |
| **61** | **s436** | AA |
| **62** | **s437** | AA |
| **63** | **s438** | AA |
| **64** | **s439** | AA |
| **65** | **s440** | AA |
| **66** | **s441** | AA |
| **67** | **s442** | AA |
| **68** | **s443** | AA |
| **69** | **s445** | AA |
| **70** | **s446** | AA |
| **71** | **s449** | AA |
| **72** | **s450** | AA |
| **73** | **s453** | AA |
| **74** | **s454** | AA |
| **75** | **s455** | AA |
| **76** | **s457** | AA |
| **77** | **s458** | AA |
| **78** | **s459** | AA |
| **79** | **s461** | AA |
| **80** | **s462** | AA |
| **81** | **s463** | AA |
| **82** | **s464** | AA |
| **83** | **s465** | AA |
| **84** | **s466** | AA |
| **85** | **s473** | AA |
| **86** | **s476** | AA |
| **87** | **s004** | AA |
| **88** | **s007** | Aa |
| **89** | **s105** | Aa |
| **90** | **s108** | Aa |
| **91** | **s114** | Aa |
| **92** | **s122** | Aa |
| **93** | **s131** | Aa |
| **94** | **s134** | Aa |
| **95** | **s137** | Aa |
| **96** | **s139** | Aa |
| **97** | **s143** | Aa |
| **98** | **s144** | Aa |
| **99** | **s148** | Aa |
| **100** | **s201** | Aa |
| **101** | **s207** | Aa |
| **102** | **s320** | Aa |
| **103** | **s322** | Aa |
| **104** | **s400** | Aa |
| **105** | **s401** | Aa |
| **106** | **s404** | Aa |
| **107** | **s405** | Aa |
| **108** | **s406** | Aa |
| **109** | **s408** | Aa |
| **110** | **s414** | Aa |
| **111** | **s416** | Aa |
| **112** | **s417** | Aa |
| **113** | **s428** | Aa |
| **114** | **s432** | Aa |
| **115** | **s444** | Aa |
| **116** | **s452** | Aa |
| **117** | **s479** | Aa |
| **118** | **s005** | aa |
| **119** | **s129** | aa |
| **120** | **s402** | aa |
| **121** | **s407** | aa |
| **122** | **s409** | aa |

**Supplementary Table 4 The detailed information of genomes of the species used in the comparison genome**

| **species** | **Download links** | **References** |
| --- | --- | --- |
| *Oryza sativa* | <ftp://ftp.ncbi.nlm.nih.gov/genomes/all/GCF/001/433/935/GCF_001433935.1_IRGSP-1.0> | Kawahara, Y. et al. Improvement of the *Oryza sativ*a Nipponbare reference genome using next generation sequence and optical map data. *Rice (N Y)*. 6, 4 (2013) |
| *Papaver somniferum* | <ftp://ftp.ncbi.nlm.nih.gov/genomes/all/GCF/003/573/695/GCF_003573695.1_ASM357369v1> | Guo, L. et al. The opium poppy genome and morphinan production. *Science*, **6412**, 343-347 (2008). |
| *Citrus sinensis* | <ftp://ftp.ncbi.nlm.nih.gov/genomes/all/GCF/000/317/415/GCF_000317415.1_Csi_valencia_1.0> | Xu, Q. et al. The draft genome of sweet orange (*Citrus sinensis*). *Nat Genet*. **45,** 59-66 (2013). |
| *Arabidopsis thaliana* | <ftp://ftp.ncbi.nlm.nih.gov/genomes/all/GCF/000/001/735/GCF_000001735.4_TAIR10.1/> | Tabata, S. et al. Sequence and analysis of chromosome 5 of the plant Arabidopsis thaliana. *Nature*. **408**, 823-826 (2000). |
| *Theobroma cacao* | <ftp://ftp.ncbi.nlm.nih.gov/genomes/all/GCF/000/208/745/GCF_000208745.1_Criollo_cocoa_genome_V2> | Argout, X. et al. The genome of Theobroma cacao. *Nature genetics.* **43**, 101-108(2011). |
| *Rosa chinensis* | <ftp://ftp.ncbi.nlm.nih.gov/genomes/all/GCA/002/994/745/GCA_002994745.2_RchiOBHm-V2> | Saint-Oyant, L. H. et al. A high-quality genome sequence of Rosa chinensis to elucidate ornamental traits. *Nature plants*. **4**, 473-84 (2013). |
| *Medicago truncatula* | <ftp://ftp.ncbi.nlm.nih.gov/genomes/all/GCF/000/219/495/GCF_000219495.3_MedtrA17_4>.0 | Tang, H. et al. An improved genome release (version Mt4.0) for the model legume Medicago truncatula. *BMC Genomics*. **15**, 312 (2014). |
| *Vitis vinifera* | <ftp://ftp.ncbi.nlm.nih.gov/genomes/all/GCF/000/003/745/GCF_000003745.3_12X> | Jaillon, O. et al. Consortium for Grapevine Genome Characterization. The grapevine genome sequence suggests ancestral hexaploidization in major angiosperm phyla. *Nature.* **449**, 463-467 (2014). |
| *Camellia sinensis* | <ftp://ftp.ncbi.nlm.nih.gov/genomes/all/GCF/004/153/795/GCF_004153795.1_AHAU_CSS_1> | Wei, C. et al. Draft genome sequence of *Camellia sinensis* var. sinensis provides insights into the evolution of the tea genome and tea quality. *Proceedings of the National Academy of Sciences*.**115**, E4151-8 (2018). |
| *Artemisia annua* | <ftp://ftp.ncbi.nlm.nih.gov/genomes/all/GCA/003/112/345/GCA_003112345.1_ASM311234v1/> | Shen, Q. et al. The Genome of Artemisia annua Provides Insight into the Evolution of Asteraceae Family and Artemisinin Biosynthesis. *Mol Plant.* **11**, 776-788 (2018). |
| *Olea europaea* | <ftp://ftp.ncbi.nlm.nih.gov/genomes/all/GCF/002/742/605/GCF_002742605.1_O_europaea_v1/> | Unver, T. et al. Genome of wild olive and the evolution of oil biosynthesis. *Proc Natl Acad Sci USA,* **114**, E9413-E9422 (2017). |
| *Sesamum indicum* | <https://www.ncbi.nlm.nih.gov/genome/genomes/11560>/ | Zhang, H. et al. Genome sequencing of the important oilseed crop *Sesamum indicum* L. *Genome Biol.***14**, 401 (2013). |

**
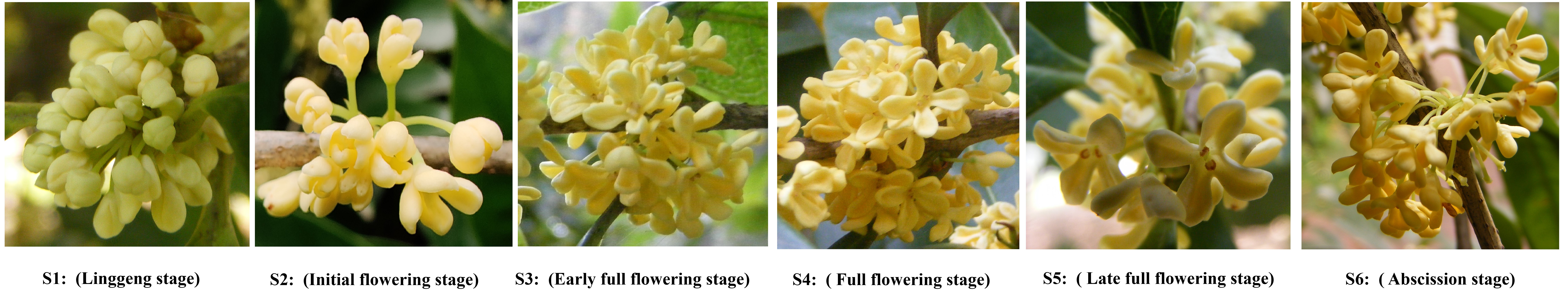
**

**Supplementary Figure 1 Developmental stages of *Osmanthus fragrans* flowers**

**(A) Stage 1 (Linggeng stage: the flower bud is closed); (B) stage 2 (Initial flowering stage: the flower slightly opened with the angle less than 45°); (C) stage 3 (Early full flowering stage: the flower half opened with the angle from 45° to 90° ); (D) stage 3 (Full flowering stage: the petals are fully expanded); (D) stage 4 (Late full flowering stage: the petals lose water slightly and the pollen becomes darker ); (E) stage 6 (Abscission stage: the petals lose water and fall off naturally).**

**
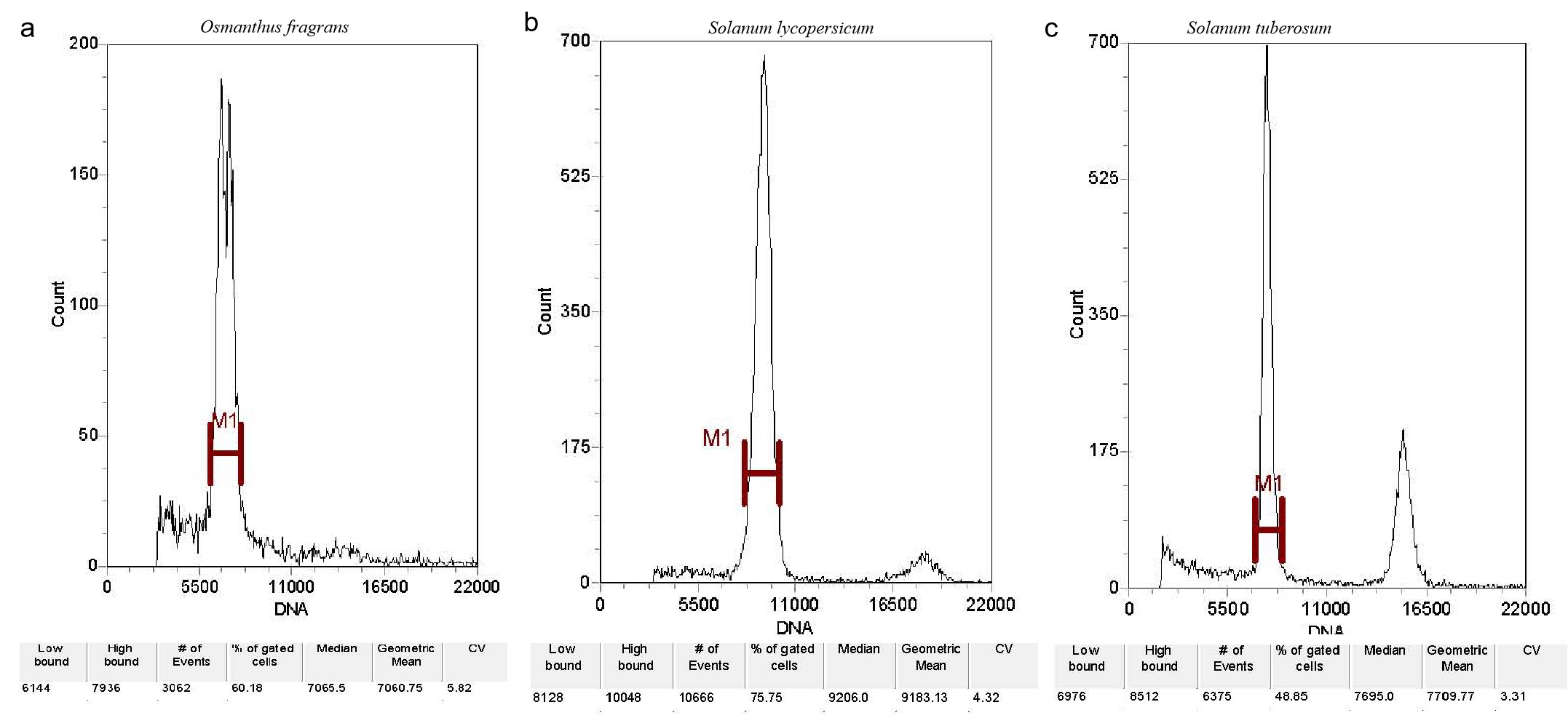
**

**Supplementary Figure 2 Flow cytometry analysis of the genome size of *Osmanthus fragrans***

**a. *Osmanthus fragrans*; b. *Solanum lycopersicum* with the genome size of 900M; c. *Solanum tuberosum* with the genome size of 844M.**

**
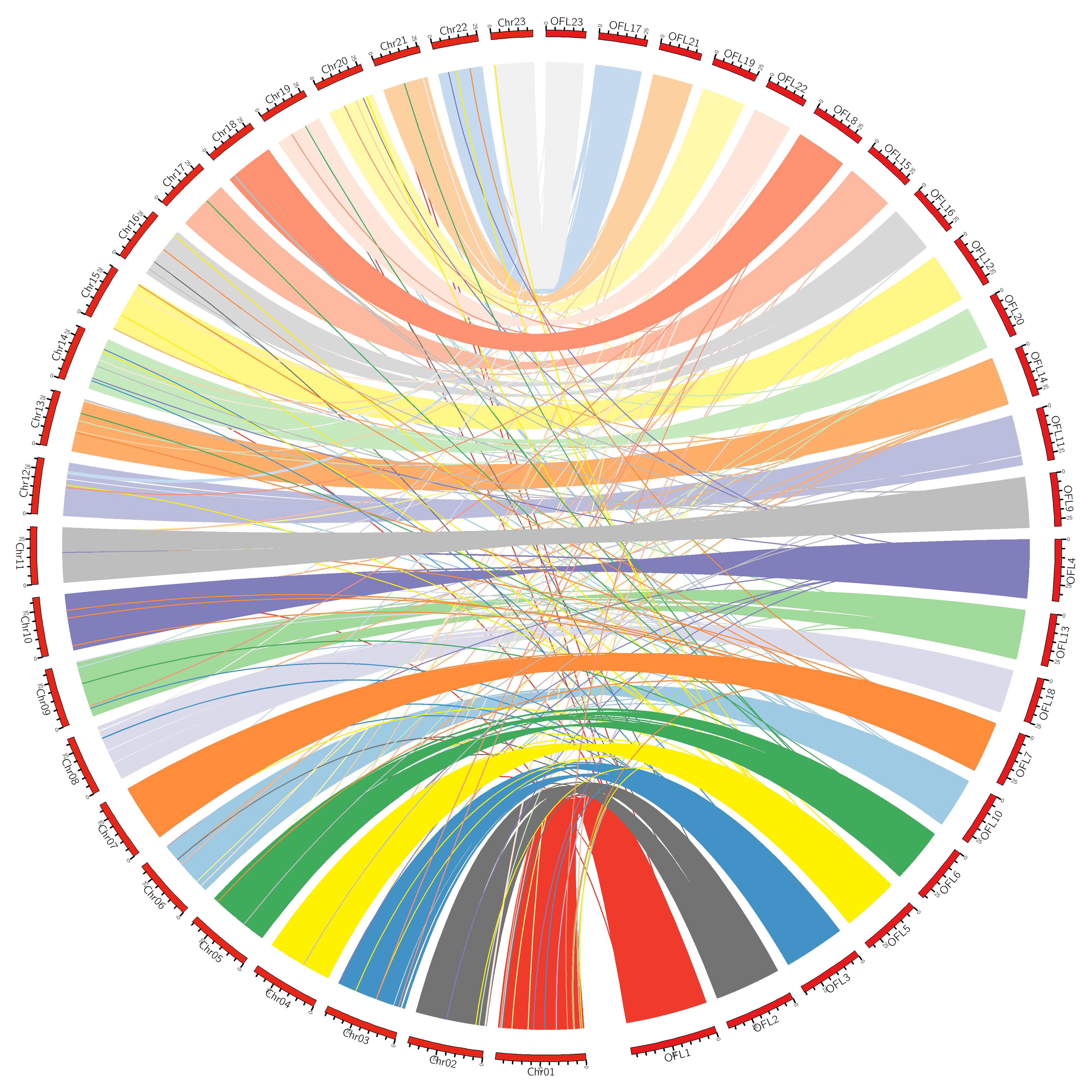
**

**Supplementary Figure 3 The Synteny analyses of the *O. fragrans* ‘Liuyejingui’ (OFL1-23) and *O. fragrans* ‘Rixianggui’ (Chr01-23)**
